# Hyperbaric oxygen therapy in sepsis-associated encephalopathy: pathophysiological rationale, preclinical evidence, and translational challenges

**DOI:** 10.3389/fncel.2026.1941147

**Published:** 2026-09-24

**Authors:** Yikun Xie, Yihao Li, Yujing Li, Shuai Chen, Guanqi Song, Hongyu Wang

**Affiliations:** The Fifth Clinical Medical College of Henan University of Chinese Medicine (Zhengzhou People's Hospital), Zhengzhou, China

**Keywords:** blood–brain barrier, ferroptosis, hyperbaric oxygen therapy, neuroprotection, oxidative stress, pyroptosis, sepsis-associated encephalopathy

## Abstract

Sepsis-associated encephalopathy (SAE) is a serious neurological complication of sepsis that can cause acute cerebral dysfunction and long-term cognitive impairment. Its pathogenesis involves multiple pathological processes, including blood–brain barrier (BBB) disruption, neuroinflammation, microglial and astrocytic responses, oxidative stress, mitochondrial dysfunction, ferroptosis, and pyroptosis. Excessive reactive oxygen species (ROS) production and mitochondrial injury can aggravate cerebrovascular endothelial and BBB damage, promote glial inflammatory responses, and contribute to lipid peroxidation and inflammasome activation, thereby linking neuroinflammation with regulated cell death. Hyperbaric oxygen therapy (HBOT) increases tissue oxygen tension and may exert neuroprotective effects by improving microcirculation and regulating redox homeostasis and inflammatory responses. Available animal studies of SAE suggest that HBOT may attenuate certain aspects of BBB injury and hippocampal neuroinflammation and improve neurobehavioral and cognitive outcomes. Findings from systemic sepsis and other neurological injury models further suggest that antioxidant responses, mitochondrial homeostasis, and signaling pathways associated with ferroptosis and pyroptosis may contribute to these effects. This review summarizes the cellular and molecular processes potentially affected by HBOT within the major pathological mechanisms of SAE and discusses its therapeutic potential and translational challenges based on the available direct and supportive evidence. Current evidence remains predominantly preclinical, and the mechanisms and clinical value of HBOT in SAE require further investigation.

## 1 Introduction

Sepsis is life-threatening organ dysfunction caused by a dysregulated host response to infection (Singer et al., 2016), and approximately 53–68% of patients with sepsis may develop sepsis-associated encephalopathy (SAE) (Sonneville et al., 2017; Lu et al., 2023). SAE is a severe neurological syndrome that usually occurs in the absence of direct central nervous system infection or another primary neurological disorder and is characterized by diffuse cerebral dysfunction caused by sepsis (Gofton and Young, 2012; Mazeraud et al., 2020). SAE may be the first manifestation of sepsis and may occur before patients are admitted to a general ward or intensive care unit (ICU) (Gofton and Young, 2012; Helbing et al., 2018). It has a high incidence and poor prognosis and substantially affects cognitive function and long-term survival.

As a major complication of sepsis, SAE encompasses a broad spectrum of cerebral dysfunction (Gofton and Young, 2012; Li J. et al., 2025). Its clinical manifestations include impaired consciousness, disorientation, cognitive dysfunction, seizures, and deep coma (Chen et al., 2020a). In children, SAE may also present with cognitive impairment, behavioral abnormalities, persistent neurological deficits, and memory impairment. Severe cases may be associated with neurodevelopmental delay, reduced verbal intelligence, poor academic performance, and intellectual decline (Kaur et al., 2016; Dumbuya et al., 2023). The clinical presentation of SAE is highly heterogeneous. Based on clinical features such as hypoxia and metabolic abnormalities, SAE has been classified into ischemic–hypoxic, metabolic, mixed, and unclassified phenotypes. Clinical outcomes differ among these phenotypes, with the mixed phenotype carrying the highest risk of in-hospital mortality (Lu et al., 2023).

Although understanding of SAE has improved, no specific treatment is currently available. Management remains focused on treating sepsis and controlling systemic inflammation to prevent or limit neurological complications. Clinical care mainly includes infection control, fluid resuscitation, vasoactive therapy, hemodynamic optimization, correction of metabolic abnormalities, and organ support (Prescott and Angus, 2018; Mazeraud et al., 2020; Sonneville et al., 2023; Li J. et al., 2025). These measures primarily address sepsis itself and secondary brain injury rather than the specific pathological processes of SAE.

The pathogenesis of SAE is complex and may result from interactions among cerebral inflammatory and ischemic injury, microcirculatory dysfunction, BBB disruption, and microglial activation (Mazeraud et al., 2020). These pathological processes jointly cause brain injury while also providing potential targets for intervention. In this context, hyperbaric oxygen therapy (HBOT), which involves breathing 100% oxygen at pressures greater than 1 atmosphere absolute to increase blood and tissue oxygen tension (Gottfried et al., 2021), may influence hypoxia, microcirculatory dysfunction, and inflammatory brain injury in SAE. Previous studies in traumatic brain injury, stroke, spinal cord injury, and neurodegenerative disorders have shown that HBOT can modulate tissue oxygenation, inflammatory responses, and neurological function (Thom, 2011; Ahmadi and Khalatbary, 2021). However, studies directly evaluating HBOT in SAE remain limited, and its proposed effects on ferroptosis, pyroptosis, and several cellular signaling pathways are based mainly on findings from other disease models.

This review examines the cellular and molecular mechanisms of SAE and analyzes the potential effects of HBOT on BBB injury, neuroinflammation, oxidative stress, mitochondrial dysfunction, ferroptosis, and pyroptosis. Available evidence is discussed at three levels: direct animal studies of SAE, studies of systemic sepsis, and studies of other neurological disorders. On this basis, current evidence gaps and translational challenges are summarized to provide a reference for future mechanistic studies and clinical evaluation of HBOT in SAE.

### 1.1 Literature search

To improve the transparency of this narrative review, we searched PubMed and Web of Science Core Collection from database inception to 31 August 2026 using combinations of the terms “sepsis-associated encephalopathy,” “sepsis,” “hyperbaric oxygen therapy,” “blood–brain barrier,” “neuroinflammation,” “microglia,” “oxidative stress,” “ferroptosis,” and “pyroptosis.” Reference lists of relevant reviews and original articles were also screened manually. Priority was given to studies directly evaluating HBOT in experimental SAE; studies of systemic sepsis and other neurological injury models were included only as supportive mechanistic evidence and are identified as such throughout the text. This review was narrative in nature and was not intended as a systematic review or meta-analysis.

## 2 Major pathological mechanisms of SAE and the rationale for HBOT

The pathogenesis of SAE is complex and involves abnormalities in cerebral perfusion and cerebral blood flow regulation, blood–brain barrier (BBB) injury, neuroinflammation and microglial activation, oxidative stress, mitochondrial dysfunction, and neurotransmitter disturbances (Bozza et al., 2013; Sharshar et al., 2014; Michels et al., 2015; Tian et al., 2019; Ren et al., 2020; Gao and Hernandes, 2021; Guo et al., 2024). These mechanisms do not act independently. Increased BBB permeability facilitates the entry of peripheral inflammatory signals into the brain and induces microglial and astrocytic responses. Inflammatory mediators and reactive oxygen species (ROS) further aggravate endothelial and mitochondrial injury and contribute to regulated cell death, including ferroptosis and pyroptosis, ultimately impairing synaptic function and neuronal survival. Thus, altered consciousness, cognitive decline, and long-term neurological impairment in SAE result from the interaction of multiple pathological processes.

Hyperbaric oxygen therapy (HBOT) involves the intermittent inhalation of 100% oxygen at pressures greater than 1 atmosphere absolute, thereby increasing arterial and tissue oxygen tension (Grim et al., 1990; Tibbles and Edelsberg, 1996). According to Henry’s law, the amount of gas dissolved in a liquid is proportional to its partial pressure. Under hyperbaric conditions, the amount of physically dissolved oxygen in plasma increases substantially, allowing oxygen to diffuse into hypoperfused or hypoxic tissues without being entirely dependent on hemoglobin transport (Lindenmann et al., 2021). Increased dissolved oxygen can improve tissue oxygenation and influence microcirculation, mitochondrial energy metabolism, and tissue repair (De Wolde et al., 2021; Schottlender et al., 2021; Lindenmann et al., 2022). These effects provide the physiological basis for the use of HBOT in ischemic, hypoxic, and inflammation-related disorders.

The effects of HBOT are not limited to increasing oxygen delivery. The transient increase in ROS and reactive nitrogen species (RNS) during treatment can act as a signaling stimulus that induces endogenous antioxidant responses and modulates mitochondrial function and inflammatory signaling (De Wolde et al., 2021; Schottlender et al., 2021). However, when the treatment pressure is excessive, oxygen exposure is prolonged, or the interval between sessions is insufficient, ROS and RNS production may exceed the capacity of endogenous antioxidant systems and aggravate oxidative injury. The biological effects of HBOT may therefore vary according to treatment pressure, exposure duration, number of sessions, and disease stage. In view of the pathological features of SAE, HBOT may act on BBB injury, neuroinflammation, oxidative stress, and regulated cell death. However, studies directly evaluating HBOT in SAE remain limited, and some proposed mechanisms are based on findings from other neurological injury models. These mechanisms are discussed below.

### 2.1 Blood–brain barrier injury and the effects of HBOT

The structural basis of the blood–brain barrier (BBB) consists primarily of brain microvascular endothelial cells, the basement membrane, pericytes, and astrocytic endfeet. Together with adjacent microglia and neurons, these components form the neurovascular unit. Neighboring endothelial cells are connected by tight junction proteins, including occludin, claudin-5, and zonula occludens-1 (ZO-1), forming a highly selective interface between the cerebral circulation and brain parenchyma. By regulating the exchange of ions, fluids, and nutrients between the blood and brain and facilitating the clearance of metabolic waste, the BBB maintains homeostasis of the cerebral microenvironment (Abbott et al., 2006).

During sepsis, lipopolysaccharide (LPS) can bind to Toll-like receptor 4 (TLR4) on brain microvascular endothelial cells and microglia and activate NF-κB signaling through a MyD88-dependent pathway. Circulating TNF-α, IL-1β, and IL-6 can also act on cerebrovascular endothelial cells and further amplify the inflammatory response, leading to reduced expression or abnormal distribution of occludin, claudin-5, and ZO-1 and consequently increasing BBB permeability (Peng et al., 2021; Song and Song, 2025). In addition to inflammatory mediators, nitric oxide signaling in cerebrovascular endothelial cells may become dysregulated during the early stage of sepsis. *In vitro* studies have shown that septic plasma increases endothelial nitric oxide synthase-derived nitric oxide and oxidant production in cerebrovascular endothelial cells, resulting in the dissociation of occludin from the cytoskeleton and increased permeability of the endothelial monolayer (Handa et al., 2008). The inflammatory response in cerebrovascular endothelial cells may precede detectable microglial activation, indicating that endothelial cells are among the earliest central nervous system targets of peripheral inflammation (Kodali et al., 2021).

Excessive ROS can also activate matrix metalloproteinases, particularly MMP-2 and MMP-9, leading to the degradation of tight junction proteins and the vascular basement membrane (Kim et al., 2024). LPS promotes the translocation of dynamin-related protein 1 (Drp1) to mitochondria in brain microvascular endothelial cells and enhances Drp1/Fis1-mediated mitochondrial fission. This process reduces the mitochondrial membrane potential, promotes ROS accumulation, and disrupts energy metabolism, ultimately causing tight junction loss and increased endothelial permeability (Haileselassie et al., 2020).

Once the BBB is disrupted, peripheral inflammatory mediators, immune cells, and other potentially toxic molecules can more readily enter the brain and activate microglia and astrocytes. Activated glial cells continue to release inflammatory mediators and ROS, further aggravating endothelial injury and establishing a self-reinforcing cycle between BBB disruption and neuroinflammation (Gao and Hernandes, 2021; Peng et al., 2021; Liu et al., 2025; Song and Song, 2025).

Endothelial dysfunction, altered sphingolipid metabolism, and mitochondrial injury also contribute to BBB disruption. For example, triggering receptor expressed on myeloid cells 1 (TREM-1) in endothelial cells may participate in sepsis-induced BBB disruption and cognitive impairment through PI3K/Akt-related signaling (Su et al., 2025). BBB injury therefore not only provides a route through which peripheral inflammatory signals enter the brain but also directly affects neuronal function, synaptic plasticity, and cognition. It represents an important pathological basis of neurological and cognitive injury in SAE and a potential target for interventions aimed at suppressing inflammation and maintaining BBB stability.

Studies examining the effects of HBOT on the BBB remain limited, and their findings are not entirely consistent. HBOT has been reported to reduce BBB hyperpermeability and cerebral edema following cerebral ischemia or reperfusion injury (Veltkamp et al., 2005; Li et al., 2018). Li et al. (2018) found that HBOT increased the expression of ZO-1 and occludin, suggesting that it may attenuate BBB injury by regulating transcellular transport and maintaining tight junctions. In an *in vitro* model of brain microvascular endothelial cells, HBOT preconditioning attenuated the hypoxia-induced reduction in transendothelial electrical resistance and increase in permeability, accompanied by the restoration of tight junction protein expression (Hao et al., 2016). Veltkamp et al. reported that HBOT suppressed the increase in plasma MMP-9 after cerebral ischemia and reduced the degradation of laminin-5 in the cerebral microvascular basement membrane (Veltkamp et al., 2006). These findings suggest that HBOT may protect the BBB by attenuating oxidative stress, inhibiting MMP activation, and preserving tight junction integrity. However, these mechanisms have mainly been identified in non-septic models.

Among studies directly evaluating HBOT in animal models of SAE, Wang Y. et al. (2024) administered HBOT at 1 and 4 h after cecal ligation and puncture (CLP; 1.9 ATA, 100% oxygen, 90 min per session). HBOT reduced HIF-1α and inflammatory mediator levels, attenuated microglial activation and BBB hyperpermeability, and improved learning and memory. In contrast, Avtan et al. (2011) administered four HBOT sessions to rats at 1, 7, 13, and 19 h after CLP using 2.5 ATA, with 60 min of oxygen exposure at the target pressure. Although HBOT reduced oxidative stress and Evans blue extravasation, it did not improve horseradish peroxidase extravasation or occludin expression and increased horseradish peroxidase extravasation in healthy rats. The differences between these studies may be related to animal species, HBOT protocols, and the methods used to assess BBB injury.

### 2.2 Neuroinflammation, microglial activation, and the effects of HBOT

Neuroinflammation refers to inflammatory responses within the central nervous system (CNS), characterized mainly by glial activation and increased production of inflammatory mediators (Moraes et al., 2021). Under physiological conditions, peripheral cytokines can affect the CNS through specific transport mechanisms, signaling across cerebrovascular endothelial cells, and neural afferent pathways. These mechanisms allow the CNS to sense peripheral inflammation and regulate systemic immune responses through neuroimmune pathways (Erickson and Banks, 2018). During sepsis, persistently elevated TNF-α, IL-1β, and IL-6 act on cerebrovascular endothelial cells, promote adhesion molecule expression, and disrupt the BBB. As BBB permeability increases, peripheral inflammatory mediators and immune cells gain greater access to brain tissue, further activating microglia and astrocytes and amplifying neuroinflammation (Erickson and Banks, 2018; Moraes et al., 2021). BBB injury therefore represents both an important route for transmitting peripheral inflammatory signals to the CNS and a basis for the persistence of neuroinflammation.

Neuroinflammation contributes to both the development of SAE and long-term cognitive impairment in survivors (Michels et al., 2015; Manabe and Heneka, 2022). This process is not mediated by a single cell type but involves interactions among cerebrovascular endothelial cells, microglia, astrocytes, and neurons (Erickson and Banks, 2018; Zhu et al., 2024). Cerebrovascular endothelial cells sense circulating inflammatory signals and disrupt BBB homeostasis. Astrocytic endfeet contribute to BBB maintenance, while activated astrocytes produce cytokines and chemokines. Injured neurons release damage-associated molecular patterns, including ATP and mitochondrial DNA, which further activate surrounding immune cells (Erickson and Banks, 2018; Moraes et al., 2021; Zhu et al., 2024). As resident immune cells of the CNS, microglia rapidly respond to these changes and play a central role in neuroinflammation during SAE (Michels et al., 2015). Postmortem studies have confirmed microglial activation in patients with sepsis, although its regional distribution has not been consistent. Lemstra et al. (2007) found a marked increase in CD68-positive microglia in the cerebral gray matter of patients with sepsis. In contrast, Zrzavy et al. (2019) reported that pro-inflammatory microglial responses were more prominent in white matter, as indicated by increased expression of CD68, CD86, HLA-DR, and inducible nitric oxide synthase, whereas changes in gray matter were less pronounced. These findings suggest that microglial responses in SAE may vary among brain regions and between white and gray matter.

Activated microglia have traditionally been classified into pro-inflammatory M1 and anti-inflammatory M2 phenotypes. IFN-γ and LPS induce the expression of pro-inflammatory molecules such as inducible nitric oxide synthase, CD86, and TNF-α, whereas IL-4 and IL-13 induce molecules associated with anti-inflammatory responses and tissue repair, including arginase 1, CD206, and IL-10 (Wolf et al., 2017; Li and Barres, 2018). However, the M1/M2 classification was derived mainly from experiments using single stimuli under *in vitro* conditions. Microglia *in vivo* do not exist as two clearly separated subtypes but instead exhibit overlapping response states that vary according to the type of stimulus, disease stage, and brain region (Ransohoff, 2016; Paolicelli et al., 2022). Single-cell studies have identified homeostatic, inflammatory, interferon-responsive, proliferative, and phagocytic microglial states (Masuda et al., 2019; Paolicelli et al., 2022). In an LPS-induced model of SAE, distinct microglial subsets also showed specific spatial associations with astrocytes and vascular cells (Zhu et al., 2024). These changes contribute to the amplification of inflammatory signaling, abnormal synaptic phagocytosis, and cognitive impairment.

During SAE, microglia recognize pathogen-associated and damage-associated molecular patterns mainly through pattern recognition receptors (Moraes et al., 2021). LPS does not need to cross an intact BBB in large amounts to induce neuroinflammation. It can act on cerebrovascular endothelial cells, the choroid plexus, and circumventricular organs to transmit peripheral inflammatory signals to the brain. Once the BBB is disrupted, LPS and other inflammatory mediators entering the brain may further enhance microglial activation (Gofton and Young, 2012; Erickson and Banks, 2018). TLR2 and TLR4 are important pattern recognition receptors in sepsis. TLR4 primarily recognizes LPS from Gram-negative bacteria, whereas TLR2 recognizes bacterial lipoproteins, peptidoglycan, and related components (Kawai and Akira, 2010; Lorne et al., 2010).

Binding of LPS to the TLR4–MD2–CD14 receptor complex recruits MyD88 and subsequently activates IRAK1/4, TRAF6, and TAK1. TAK1 activates the IKK complex, promoting the phosphorylation and degradation of IκBα and allowing NF-κB to translocate into the nucleus. It also activates the p38, JNK, and ERK mitogen-activated protein kinase pathways. Together, these signals promote the expression of TNF-α, IL-1β, IL-6, chemokines, and inducible nitric oxide synthase (Kawai and Akira, 2010; Moraes et al., 2021; Manabe and Heneka, 2022). ROS, extracellular ATP, and mitochondrial DNA generated during inflammation can further promote NLRP3 inflammasome assembly, caspase-1 activation, and the maturation and release of IL-1β and IL-18 (Moraes et al., 2021, 2023; Manabe and Heneka, 2022).

Moraes et al. (2023) found that CLP increased the expression of NLRP3 inflammasome-related molecules in hippocampal microglia. In primary microglia stimulated with LPS, mitochondrial ROS promoted NLRP3 inflammasome assembly and IL-1β release, whereas caspase-1-dependent microvesicles enriched in IL-1β caused neurite injury and excitatory synapse loss. TLR4/NF-κB signaling therefore provides the priming signal for the expression of NLRP3 and pro-IL-1β, while ROS, extracellular ATP, and mitochondrial DNA promote inflammasome assembly and caspase-1 activation. These processes jointly amplify microglia-mediated neuroinflammation and synaptic injury.

Microglial activation affects neurological function not only through inflammatory mediator release but also through complement-mediated abnormal synaptic phagocytosis. During normal neural development, microglia engulf selected synaptic structures and contribute to synaptic pruning and neural circuit formation (Paolicelli et al., 2011). During sepsis, excessive complement activation causes abnormal C1q deposition on excitatory synapses, which are subsequently recognized and engulfed by microglia. Chung et al. (2023) observed C1q deposition, synaptic loss, and increased microglial phagocytosis in the hippocampi of patients with sepsis and mice with polymicrobial sepsis. Microglial depletion or C1q blockade reduced neuronal and synaptic injury and improved cognitive function. Increased C1q expression, enhanced microglial phagocytosis, and excitatory synapse loss have also been observed in a neuroinflammation model induced by repeated LPS administration (Wu et al., 2023).

Administration of exogenous cholecystokinin or activation of endogenous cholecystokinin-positive neurons reduced C1q labeling of excitatory synapses, suppressed microglial phagocytosis, and improved cognitive function in mice with SAE (Chen et al., 2025). Resolvin D1 attenuated microglia-mediated neuroinflammation by inhibiting NF-κB signaling (Xu et al., 2022). Our previous study also showed that Foxc1 overexpression suppressed microglial migration, inflammatory responses, and neuronal apoptosis through regulation of the IκBα/NF-κB pathway, thereby ameliorating SAE-associated cognitive impairment (Wang H. et al., 2022). These findings indicate that microglia not only amplify inflammatory signaling during the early stage of SAE but may also impair learning and memory through abnormal synaptic engulfment.

Studies in other neurological injury models provide supportive evidence that HBOT can regulate microglial responses. In a traumatic brain injury model, HBOT increased IL-10 levels in brain tissue, whereas IL-10 blockade weakened its anti-inflammatory and neuroprotective effects (Chen X. et al., 2014). Qian et al. (2017) found that HBOT reduced the expression of NLRP3, ASC, and caspase-1 in the perilesional cortex and decreased IL-1β and IL-18 release. Immunofluorescence analysis showed that NLRP3 was predominantly localized in microglia. HBOT also reduced microglial activation and neuroinflammation in 3xTg-AD mice (Shapira et al., 2018). In addition, early HBOT improved survival and regulated inflammatory mediator expression in liver tissue and macrophages in mice with severe sepsis (Halbach et al., 2019), although brain injury and cognitive function were not assessed. These findings suggest that IL-10 and the NLRP3 inflammasome may be involved in the regulation of neuroinflammation by HBOT.

Direct evidence regarding the effects of HBOT on neuroinflammation in SAE remains limited. Wang Y. et al. (2024) administered HBOT at 1 and 4 h after CLP in mice using 1.9 ATA and 100% oxygen for 90 min per session. HBOT reduced HIF-1α, TNF-α, IL-1β, and IL-6 levels in blood and hippocampal tissue, attenuated hippocampal microglial activation and BBB hyperpermeability, and improved learning and memory. Hippocampal HIF-1α overexpression weakened these effects. These findings suggest that HBOT may attenuate brain injury in SAE by regulating HIF-1α-associated inflammatory responses. However, because HIF-1α was not manipulated in a specific cell type, its direct relationship with microglial responses requires further investigation.

### 2.3 Oxidative stress, ferroptosis, and pyroptosis in SAE and their potential modulation by HBOT

#### 2.3.1 Oxidative stress and its modulation by HBOT

Neuroinflammation persists throughout the development of SAE, accompanied by oxidative stress and regulated cell death. Oxidative stress occurs during the early stage of sepsis and can reduce intracellular ATP production and induce endoplasmic reticulum stress and neuronal injury (Nagar et al., 2018; Li et al., 2020; Zhang et al., 2025). During sepsis, inflammatory mediator release, NADPH oxidase activation, mitochondrial dysfunction, and impaired endogenous antioxidant capacity collectively promote the accumulation of reactive oxygen species (ROS) and reactive nitrogen species, resulting in sustained redox imbalance (Sahoo et al., 2024; Yang et al., 2026).

Cerebrovascular endothelial cells and activated microglia generate ROS through NADPH oxidases and damaged mitochondria. Increased inducible nitric oxide synthase expression also leads to excessive nitric oxide production. Nitric oxide competes with oxygen for binding to cytochrome c oxidase and thereby inhibits mitochondrial respiration. Its reaction with superoxide produces peroxynitrite, which damages respiratory chain complexes I and IV, reduces the mitochondrial membrane potential, and impairs ATP production (Hubbard et al., 2004; Poderoso et al., 2019). Damaged mitochondria subsequently release additional ROS, further aggravating oxidative injury.

The consequences of oxidative stress vary among different brain cell types. Injury to cerebrovascular endothelial cells disrupts tight junctions and increases BBB permeability. ROS accumulation in microglia promotes NF-κB signaling and NLRP3 inflammasome activation. Impaired metabolic and antioxidant support from astrocytes reduces neuronal resistance to oxidative injury, whereas neurons are particularly susceptible to mitochondrial dysfunction and lipid peroxidation because of their high energy demands (Schwalm et al., 2014; Gu et al., 2021). In rats surviving sepsis, oxidative brain injury during the acute phase has been associated with long-term cognitive impairment and neurodegenerative changes, further supporting the involvement of oxidative stress in both acute brain injury and persistent neurological dysfunction after SAE (Schwalm et al., 2014).

HBOT has a dual effect on oxidative stress. Hyperoxic exposure initially increases tissue oxygen tension and promotes ROS and reactive nitrogen species production; therefore, HBOT is not an antioxidant treatment that directly scavenges ROS. A moderate and intermittent increase in ROS can act as a redox signal and initiate endogenous antioxidant and cellular repair responses. However, excessive treatment pressure, prolonged exposure, or insufficient intervals between sessions may cause ROS production to exceed endogenous antioxidant capacity, resulting in lipid, protein, and DNA damage (Thom, 2009). Animal studies have shown that exposure to hyperbaric oxygen at 3–5 ATA increases BBB permeability, suggesting that excessive pressure may shift the response from adaptive redox signaling toward oxidative injury (Danino et al., 2024). Whether HBOT is protective or harmful therefore depends not only on oxygen concentration but also on treatment pressure, exposure duration, treatment frequency, intervention timing, and the pre-existing redox state of the tissue.

One possible mechanism underlying the adaptive response to HBOT is activation of the nuclear factor erythroid 2-related factor 2 (Nrf2) pathway. Nrf2 is a redox-sensitive transcription factor that regulates several genes involved in reducing the cellular free-radical burden, including heme oxygenase-1 (HO-1), NAD(P)H quinone oxidoreductase 1 (NQO1), and glutathione S-transferases (Tonelli et al., 2018). Under basal conditions, Nrf2 binds to Kelch-like ECH-associated protein 1 (Keap1) and undergoes continuous degradation through the Cullin 3 ubiquitin ligase complex. When ROS or electrophiles modify cysteine residues in Keap1, Nrf2 degradation is inhibited. Stabilized Nrf2 translocates into the nucleus, forms heterodimers with small Maf proteins, and binds to antioxidant response elements. This promotes the transcription of HO-1, NQO1, glutamate-cysteine ligase catalytic subunit, and glutamate-cysteine ligase modifier subunit, thereby increasing glutathione synthesis and cellular ROS-scavenging capacity (Taguchi et al., 2011; Tonelli et al., 2018). HO-1, also known as heat shock protein 32, is an important mediator of resistance to oxidative injury and has neuroprotective effects (Chen, 2014). Activation of this pathway enhances cellular antioxidant capacity and may suppress inflammation-associated ROS overproduction (Godman et al., 2010; Taguchi et al., 2011; Verma et al., 2015; Harrison et al., 2018).

Available studies provide preliminary evidence that HBOT activates Nrf2-associated responses. In human microvascular endothelial cells, HBOT regulated the expression of Nrf2- and HMOX1-related genes and increased resistance to oxidative injury (Godman et al., 2010). In neonatal rat hypoxic–ischemic brain injury and cellular oxygen–glucose deprivation models, hyperbaric oxygen preconditioning increased Nrf2 expression and enhanced the activity of downstream antioxidant molecules, including HO-1 and glutathione S-transferase. Knockdown of Nrf2 markedly weakened its anti-apoptotic and neuroprotective effects (Zhai et al., 2016). These findings suggest that Nrf2 is involved in the antioxidant adaptation induced by HBOT. However, these results were obtained from non-cerebral microvascular endothelial cells and hypoxic–ischemic brain injury models and do not establish that HBOT acts through Nrf2 in a specific brain cell type during SAE.

In addition to Nrf2 signaling, the sirtuin 1–peroxisome proliferator-activated receptor γ coactivator 1α (SIRT1–PGC-1α) pathway may contribute to the regulation of mitochondrial homeostasis by HBOT. SIRT1 activates PGC-1α through deacetylation, which subsequently regulates downstream molecules such as nuclear respiratory factor 1 and mitochondrial transcription factor A, promotes mitochondrial biogenesis, and maintains respiratory chain function. In a neurodegenerative disease model, HBOT increased the expression of SIRT1, PGC-1α, and mitochondrial transcription factor A, promoted mitochondrial biogenesis, and attenuated neuronal injury (Hsu et al., 2022). In models of cerebral ischemia and oxygen–glucose deprivation in primary cortical neurons, hyperbaric oxygen preconditioning increased SIRT1 expression, whereas SIRT1 inhibition or knockdown markedly reduced its neuroprotective effect (Yan et al., 2013). These studies provide mechanistic evidence for the involvement of SIRT1 in HBOT-mediated neuroprotection, although this pathway has not been directly examined in SAE.

Few studies have directly examined the effect of HBOT on oxidative stress in the brain during SAE. Avtan et al. (2011) found that HBOT reduced malondialdehyde levels and restored glutathione content in the cerebral cortex and hippocampus of rats after CLP, suggesting reduced lipid peroxidation and improved antioxidant status. However, the study did not examine Nrf2, SIRT1, or mitochondrial respiratory function and did not determine whether these changes occurred predominantly in neurons, glial cells, or cerebrovascular endothelial cells. Moreover, although HBOT reduced Evans blue extravasation, it did not improve horseradish peroxidase extravasation or occludin expression. Improvements in oxidative stress markers therefore did not indicate complete recovery of BBB structure and function.

In a focal cerebral ischemia model, repeated HBOT reduced reactive nitrogen species and lipid peroxidation in brain tissue and enhanced endogenous antioxidant defenses more effectively than a single session (Chen L.-F. et al., 2014). This finding nevertheless comes from a different neurological injury model. Overall, current evidence suggests that HBOT may attenuate oxidative brain injury in SAE through Nrf2-mediated antioxidant responses and SIRT1–PGC-1α-mediated regulation of mitochondrial homeostasis. The cell types involved and the interactions and causal relationships between these pathways remain to be clarified.

#### 2.3.2 Ferroptosis and its potential modulation by HBOT

Ferroptosis is a form of regulated cell death driven by iron-dependent lipid peroxidation and is characterized by disrupted intracellular iron metabolism and excessive ROS accumulation (Dixon et al., 2012; Jiang X. et al., 2021). Under physiological conditions, iron metabolism and ROS production are balanced by antioxidant systems involving glutathione peroxidase 4 (GPX4), glutathione (GSH), and system Xc^−^, thereby maintaining cellular redox homeostasis. Under pathological conditions, the accumulation of ferrous iron promotes hydroxyl radical generation through the Fenton reaction and aggravates oxidative stress and membrane lipid peroxidation (Stockwell et al., 2017; Zhong et al., 2025). Acyl-CoA synthetase long-chain family member 4 (ACSL4) activates polyunsaturated fatty acids (PUFAs), which are subsequently incorporated into membrane phospholipids. Oxidation of these phospholipids by lipoxygenases and ROS produces phospholipid hydroperoxides (PUFA-PL-OOH), ultimately causing membrane damage and ferroptotic cell death (Doll et al., 2017; Kagan et al., 2017).

Several intracellular antioxidant systems suppress ferroptosis (Duo et al., 2025). System Xc^−^ imports cystine through solute carrier family 7 member 11 (SLC7A11) and supports GSH synthesis. GPX4 uses GSH as a reducing substrate to remove phospholipid hydroperoxides, making the SLC7A11–GSH–GPX4 axis a major defense mechanism against ferroptosis (Yang W. S. et al., 2014; Jiang X. et al., 2021). In addition, the ferroptosis suppressor protein 1–coenzyme Q10, dihydroorotate dehydrogenase–ubiquinol, and GTP cyclohydrolase 1–tetrahydrobiopterin pathways inhibit lipid peroxidation in different subcellular compartments and provide relatively independent ferroptosis defense systems (Bersuker et al., 2019; Doll et al., 2019; Kraft et al., 2020; Mao et al., 2021).

Ferroptosis in SAE is characterized by cerebral iron accumulation, increased lipid peroxidation, reduced GPX4 and SLC7A11 expression, and mitochondrial structural damage. Xie et al. (2022) found that ferrostatin-1 attenuated neuronal ferroptosis, glutamate excitotoxicity, and synaptic injury by inhibiting lipid peroxidation in CLP mice. Wang et al. (2023) further showed that ferrostatin-1 reduced BBB hyperpermeability and microglial activation and improved cognitive dysfunction in mice with SAE, accompanied by increased Nrf2/HO-1 signaling. Irisin and maresin 1 attenuated hippocampal neuronal ferroptosis and neuroinflammation through the Nrf2/GPX4 and SLC7A11/GPX4 axes, respectively (Wang J. et al., 2022; Wu et al., 2024).

Disrupted iron homeostasis also contributes to ferroptosis in SAE. Autophagy can limit neuronal iron uptake by degrading transferrin receptor 1 (TFR1) (Du et al., 2023), whereas c-MAF-mediated transcription of Slc40a1 helps maintain neuronal iron export (Song W. et al., 2026). The iron chelator deferoxamine reduces hippocampal iron accumulation, mitochondrial injury, lipid peroxidation, and inflammation, possibly through regulation of the PEBP1/15-LOX/GPX4 pathway (Wang H. et al., 2024).

Ferroptosis in SAE is not restricted to neurons. Exosomes derived from septic serum can promote ferroptosis in brain endothelial cells through the NEAT1/miR-9-5p/TFRC and GOT1 axes, potentially contributing to BBB injury (Wei et al., 2022). Activated microglia can release CXCL2 and promote neuronal ferroptosis through neuronal CXCR2/Jun signaling, indicating a direct link between microglial inflammation and neuronal lipid peroxidation (Yang Y. S. et al., 2024). Nrf2 may also suppress hippocampal ferroptosis by maintaining mitochondrial dynamic homeostasis (Duan et al., 2024). These findings indicate that ferroptosis in SAE involves interactions among brain endothelial cells, microglia, and neurons and is closely associated with BBB injury, oxidative stress, neuroinflammation, and cognitive impairment.

Recent studies have shown that HBOT can regulate ferroptosis through several molecular pathways. In a mouse model of spinal cord injury, HBOT reduced neuronal ferroptosis and improved neurological function (Yao et al., 2023). In a rat model of cerebral ischemia–reperfusion injury, HBOT reduced iron accumulation, ROS production, and lipid peroxidation and consequently attenuated brain injury (Chen et al., 2023). Further studies showed that HBOT enhanced cellular antioxidant defenses through the Nrf2/GPX4 and system Xc^−^/GPX4 axes and suppressed ferroptosis in spinal cord injury models and in HT22 and PC12 cells subjected to oxygen–glucose deprivation/reoxygenation (Chen et al., 2022; Li C. et al., 2025).

These findings were obtained mainly from spinal cord injury, cerebral ischemia–reperfusion injury, and neural cell models. Whether HBOT affects the SLC7A11–GSH–GPX4 axis, Nrf2, TFR1, Slc40a1, or microglial CXCL2 signaling in SAE has not yet been established and requires direct investigation in SAE models.

#### 2.3.3 Pyroptosis and its potential modulation by HBOT

Pyroptosis is a form of regulated cell death mediated by inflammatory caspases and gasdermin family proteins. It is characterized by progressive cell swelling, plasma membrane rupture, and the release of intracellular contents, thereby inducing a strong inflammatory response. The canonical pyroptosis pathway is mainly mediated by the NLRP3 inflammasome and generally involves priming and activation. During priming, pathogen-associated or damage-associated molecular patterns promote the expression of NLRP3, pro-IL-1β, and pro-IL-18 through signaling pathways such as TLR4/MyD88/NF-κB. Subsequent signals, including K^+^ efflux, increased ROS production, mitochondrial dysfunction, and mitochondrial DNA release, promote the assembly of NLRP3, apoptosis-associated speck-like protein containing a CARD (ASC), and pro-caspase-1 into the inflammasome complex (Swanson et al., 2019; Danielski et al., 2020; Li J. et al., 2025).

Activated caspase-1 cleaves gasdermin D (GSDMD) to generate the pore-forming GSDMD N-terminal fragment (GSDMD-NT) and simultaneously promotes the maturation of pro-IL-1β and pro-IL-18. GSDMD-NT forms pores in the plasma membrane, resulting in ionic imbalance, cell swelling, membrane rupture, and the release of IL-1β and IL-18, which further amplify inflammation (Jorgensen and Miao, 2015; Liu et al., 2016; Xia et al., 2021; Yu et al., 2021). In the non-canonical pathway, cytosolic LPS directly activates caspase-11 in mice or caspase-4/5 in humans, leading to GSDMD cleavage. The resulting GSDMD pores induce K^+^ efflux, which can further activate the NLRP3 inflammasome (Kayagaki et al., 2015; Broz et al., 2020).

Cell-specific studies of pyroptosis in SAE have mainly focused on brain endothelial cells and microglia. Inhibition of the NLRP3/caspase-1/GSDMD pathway reduces brain endothelial cell pyroptosis, BBB injury, and cognitive dysfunction (Zhou et al., 2023; Song S. et al., 2026). Song S. et al. (2026) further showed that brain endothelial cell pyroptosis was associated with increased BBB permeability and abnormal tight junction protein expression.

Microglial pyroptosis is regulated by several upstream pathways. In CLP-induced SAE, dimethyl fumarate and the STING inhibitor C-176 attenuated microglial pyroptosis, reduced the release of inflammatory mediators in the hippocampus, and improved survival and cognitive function. These findings suggest that cGAS–STING signaling activated after mitochondrial injury may act upstream of microglial pyroptosis (Zeng et al., 2025). Erbin reduced NLRP3-dependent microglial pyroptosis by inhibiting the IRE1α/XBP1s–Ca^2+^ axis (Jing et al., 2022). The protective effects of gelsevirine and the histone deacetylase 3 inhibitor RGFP966 were also associated with inhibition of the STING and STING/NLRP3 pathways, respectively (Zeng et al., 2024; Cai et al., 2026).

Pyroptosis may, in turn, aggravate mitochondrial and synaptic injury. The mitochondria-targeted peptide SS-31 improved cognitive function in mice with SAE by suppressing Drp1-mediated mitochondrial fission and NLRP3 inflammasome activation (Zhong et al., 2023). GSDMD activation promoted Drp1-associated mitochondrial injury, hippocampal synaptic damage, and abnormal neural oscillations, whereas inhibition of GSDMD or Drp1 attenuated these changes (Fu et al., 2024). Current evidence therefore indicates that pyroptosis in brain endothelial cells and microglia contributes to SAE by disrupting the BBB, amplifying inflammation, and impairing synaptic function. Whether pathologically relevant pyroptosis also occurs in neurons and astrocytes remains insufficiently defined.

HBOT has been reported to regulate the NLRP3 inflammasome and pyroptosis in several injury and inflammatory models. In carbon monoxide poisoning, HBOT suppressed NLRP3 inflammasome activation and reduced caspase-1, IL-1β, and IL-18 expression in brain tissue, accompanied by attenuation of neurological injury and cognitive dysfunction (Qi et al., 2020). In a rat model of spinal cord injury, HBOT also inhibited the NLRP3 inflammasome and its downstream inflammatory response, protected neural cells, and improved motor function (Liang et al., 2015). In a chronic wound model, HBOT reduced NLRP3, caspase-1, and GSDMD expression and attenuated pyroptotic responses (Ma et al., 2024), although this finding was obtained from non-neural tissue.

In traumatic brain injury models, HBOT suppressed NLRP3 inflammasome signaling and attenuated inflammatory brain injury (Geng et al., 2016; Qian et al., 2017). HBOT also reduced hippocampal microglial pyroptosis in a heatstroke model (Xu et al., 2024). In neural stem cells subjected to oxygen–glucose deprivation/reperfusion, HBOT attenuated pyroptosis and promoted neurogenesis through the lncRNA-H19/miR-423-5p/NLRP3 axis (Ye et al., 2022).

Some of these HBOT studies assessed changes in NLRP3, ASC, caspase-1, and inflammatory mediators without simultaneously examining GSDMD cleavage, membrane pore formation, or cell lysis. These findings therefore mainly support suppression of the inflammasome response and do not by themselves establish direct inhibition of pyroptosis. Taken together, evidence from SAE and other disease models suggests that ROS production and mitochondrial injury may represent upstream processes affected by HBOT, whereas cGAS–STING and NLRP3/caspase-1/GSDMD may be potential downstream signaling nodes. This proposed mechanism requires confirmation through cell-specific interventions in models of SAE.

### 2.4 Interactions among the major pathological mechanisms of SAE and potential targets of HBOT

SAE does not result from injury to a single pathway or cell type. Instead, it develops through interactions among BBB disruption, neuroinflammation, oxidative stress, mitochondrial dysfunction, and regulated cell death. During sepsis, circulating LPS and inflammatory mediators act on cerebrovascular endothelial cells, activate inflammatory signaling pathways such as TLR4/NF-κB, and increase ROS production. These changes reduce tight junction protein expression and increase BBB permeability (Peng et al., 2021). Transcriptomic studies have shown that inflammatory responses in cerebrovascular endothelial cells may occur before detectable microglial responses, suggesting that these cells are an important interface through which peripheral inflammation is transmitted to the CNS (Kodali et al., 2021). Following BBB disruption, inflammatory mediators and other blood-derived components enter the brain and further induce astrocytic and microglial responses. Activated glial cells release additional inflammatory mediators and ROS, which aggravate endothelial injury and BBB disruption and thereby sustain the inflammatory response (Kodali et al., 2021; Peng et al., 2021; Guo et al., 2024).

ROS accumulation and mitochondrial dysfunction link BBB injury and neuroinflammation with ferroptosis and pyroptosis. In cerebrovascular endothelial cells, excessive Drp1-mediated mitochondrial fission impairs energy metabolism and aggravates oxidative stress, resulting in tight junction disruption and BBB dysfunction (Haileselassie et al., 2020). In microglia, mitochondrial injury and cytosolic DNA sensing through cGAS–STING signaling promote NLRP3 inflammasome activation, caspase-1-dependent GSDMD cleavage, and inflammatory mediator release (Zeng et al., 2025). Neurons are particularly sensitive to impaired energy supply and lipid peroxidation. Excessive ROS can deplete GSH and reduce the capacity of GPX4 to remove lipid hydroperoxides, whereas dysregulation of the PEBP1/15-LOX/GPX4 pathway promotes ferroptosis in hippocampal neurons (Wang H. et al., 2024).

Activation of the NLRP3/caspase-1/GSDMD pathway in brain endothelial cells can induce endothelial pyroptosis and aggravate BBB injury (Song S. et al., 2026). GSDMD may also affect hippocampal synaptic structure and neural oscillations through Drp1-associated mitochondrial injury, ultimately contributing to learning and memory impairment (Fu et al., 2024). Ferroptosis and pyroptosis are therefore not independent of BBB disruption and neuroinflammation. Both are influenced by oxidative stress and mitochondrial dysfunction and contribute to SAE through barrier disruption, inflammatory amplification, and synaptic injury.

HBOT may initially affect upstream processes such as oxygen delivery, redox balance, and mitochondrial function. Moderate hyperoxic stimulation can induce endogenous antioxidant responses, improve mitochondrial energy metabolism, and increase cellular resistance to subsequent oxidative injury. In contrast, excessive ROS production may have adverse effects, which could partly explain differences among previous studies (Schottlender et al., 2021). In a mouse model of SAE, HBOT reduced HIF-1α, TNF-α, IL-1β, and IL-6 levels, attenuated hippocampal microglial activation and BBB hyperpermeability, and improved learning and memory (Wang Y. et al., 2024). Another study found that HBOT reduced cerebral malondialdehyde levels, restored GSH content, and decreased Evans blue extravasation but did not significantly improve occludin expression or other permeability measures (Avtan et al., 2011). These findings suggest that the effects of HBOT on the BBB may involve reduced oxidative stress and improvement in selected aspects of barrier function rather than complete restoration of BBB structure.

Our study similarly showed that HBOT attenuated hippocampal tissue injury, reduced serum neuron-specific enolase and S100 calcium-binding protein B levels, and improved neurological and cognitive deficits in mice with SAE (Xie et al., 2026). Together, these studies provide direct evidence that HBOT can influence oxidative stress, neuroinflammation, and BBB injury in experimental SAE.

Findings from other neurological injury models further suggest that HBOT-mediated regulation of redox balance and mitochondrial function may affect downstream ferroptotic and pyroptotic pathways. In spinal cord injury models, HBOT enhanced Nrf2/GPX4-associated antioxidant defenses and reduced lipid peroxidation and neuronal ferroptosis (Yao et al., 2023; Li C. et al., 2025). In a heatstroke model, HBOT inhibited NLRP3 inflammasome activation and pyroptosis in microglia and attenuated hippocampal inflammation and neuronal injury (Xu et al., 2024). In an oxygen–glucose deprivation model, HBOT reduced neural stem cell pyroptosis through the lncRNA-H19/miR-423-5p/NLRP3 axis (Ye et al., 2022). These findings suggest that the effects of HBOT on ferroptosis and pyroptosis may be linked through its regulation of ROS production, mitochondrial function, and inflammatory signaling.

Overall, HBOT may act at several points within the pathological network of SAE by regulating redox and mitochondrial homeostasis. It may attenuate oxidative injury and preserve BBB function in cerebrovascular endothelial cells, limit the sustained amplification of inflammatory signaling in microglia and astrocytes, enhance Nrf2/GPX4-associated antioxidant capacity and reduce lipid peroxidation in neurons, and potentially alleviate inflammatory cell injury by suppressing the NLRP3/caspase-1/GSDMD pathway. Together, these effects may reduce BBB disruption, neuroinflammation, and synaptic injury and thereby improve neurological and cognitive dysfunction associated with SAE. The interactions among these pathological processes and the potential sites at which HBOT may modulate SAE are summarized in Figure 1.

**Figure 1:**
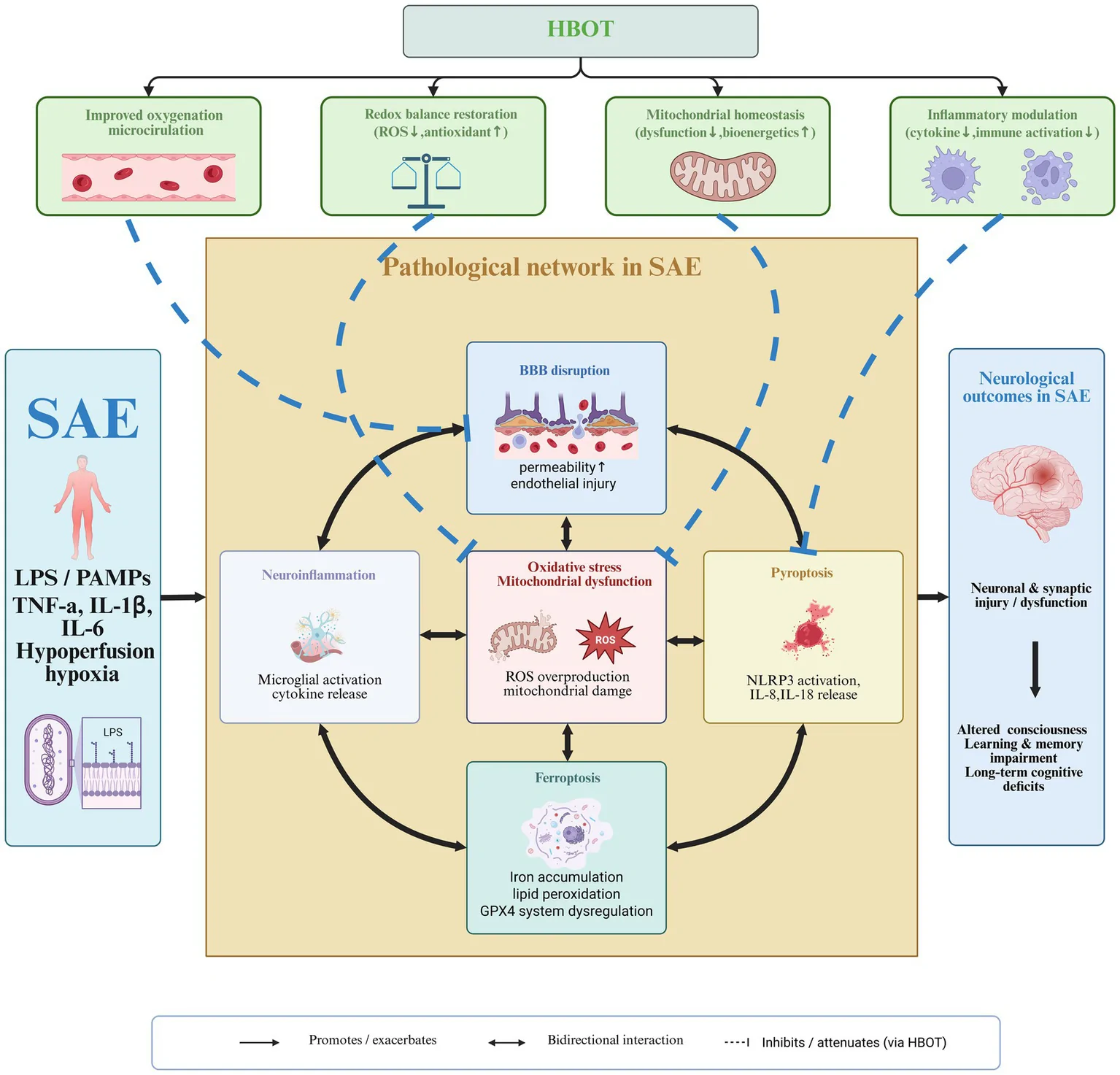
Interconnected pathological mechanisms of sepsis-associated encephalopathy and potential sites of action of hyperbaric oxygen therapy. Systemic inflammatory signals, hypoperfusion, and hypoxia contribute to blood–brain barrier (BBB) disruption, neuroinflammation, oxidative stress and mitochondrial dysfunction, ferroptosis, and pyroptosis. These processes interact with one another and ultimately promote neuronal and synaptic injury, resulting in acute cerebral dysfunction and long-term cognitive impairment. By improving oxygenation and microcirculation and modulating redox balance, mitochondrial homeostasis, and inflammatory responses, hyperbaric oxygen therapy (HBOT) may attenuate multiple nodes within this pathological network. Solid arrows indicate promoting or exacerbating effects; double-headed arrows indicate bidirectional interactions; dashed inhibitory arrows indicate potential attenuation by HBOT. BBB, blood–brain barrier; HBOT, hyperbaric oxygen therapy; IL, interleukin; LPS, lipopolysaccharide; PAMPs, pathogen-associated molecular patterns; ROS, reactive oxygen species; SAE, sepsis-associated encephalopathy; TNF-α, tumor necrosis factor-α. Created in BioRender. Tu, F. (2026) https://BioRender.com/aoh8tn1

## 3 Current evidence for HBOT in SAE

According to the study population and the relevance of the outcomes to SAE, the available evidence can be divided into three categories: studies directly examining brain injury, neurobehavioral outcomes, or cognitive function in septic animals; studies evaluating the effects of HBOT on systemic inflammation, oxidative stress, and organ injury during sepsis; and indirect evidence from traumatic brain injury, stroke, and neurodegenerative disorders. These categories address different questions. Animal studies of SAE directly evaluate the effects of HBOT on sepsis-induced brain injury. Systemic sepsis studies mainly demonstrate its biological effects within the septic environment, whereas studies of other neurological disorders indicate whether HBOT can affect pathological processes that overlap with those of SAE.

### 3.1 Direct animal studies of HBOT in SAE

Studies directly evaluating HBOT in SAE remain scarce, and no clinical studies involving patients with SAE have been reported. The available direct evidence is derived mainly from animal experiments. Avtan et al. (2011) established a rat model of sepsis using CLP and found that HBOT reduced malondialdehyde levels, restored GSH content in brain tissue, and decreased Evans blue extravasation. However, it did not significantly improve horseradish peroxidase extravasation or occludin expression. HBOT also increased horseradish peroxidase extravasation in the brains of healthy rats. The authors therefore concluded that although HBOT attenuated certain aspects of oxidative stress and altered BBB permeability, it did not comprehensively preserve BBB integrity.

Wang Y. et al. (2024) established a mouse model of SAE using CLP and administered HBOT at 1 and 4 h after surgery. HBOT reduced HIF-1α, TNF-α, IL-1β, and IL-6 levels in peripheral blood and hippocampal tissue, attenuated hippocampal microglial activation and BBB hyperpermeability, and improved learning and memory in the Barnes maze and contextual fear-conditioning tests. Hippocampal overexpression of HIF-1α weakened these effects, suggesting that HBOT may attenuate neuroinflammation and cognitive dysfunction in SAE by regulating HIF-1α-associated inflammatory responses.

Our group established a mouse model of SAE using CLP and administered HBOT at 0.20 MPa for 60 min once daily for 3, 7, or 14 days, beginning on the day after CLP. HBOT improved Morris water maze performance and SAE-associated neurobehavioral scores, attenuated hippocampal neuronal injury, and reduced serum neuron-specific enolase and S100 calcium-binding protein B levels. Some measures of neurological injury and cognitive function showed further improvement with longer treatment courses (Xie et al., 2026). This study provides experimental evidence regarding the effects of different HBOT treatment durations on cognitive function in SAE, although the underlying molecular mechanisms were not examined.

### 3.2 Supportive evidence from systemic sepsis models

In addition to direct studies of SAE, several systemic sepsis experiments have examined the effects of HBOT on inflammation, oxidative stress, and survival. Buras et al. (2006) found that HBOT increased survival in mice subjected to CLP, possibly through an IL-10-dependent anti-inflammatory response. Halbach et al. (2019) reported that HBOT initiated 1 h after CLP improved survival, whereas a single session delayed until 6 or 21 h after CLP did not produce the same effect. Bærnthsen et al. (2017) initiated HBOT 24 h after CLP and observed increased IL-10 levels at selected time points but no significant reduction in mortality. These findings suggest that the effects of HBOT in sepsis may depend on the timing of treatment and that early and delayed interventions do not necessarily produce the same outcomes.

In models of sepsis induced by LPS or bacterial infection, HBOT reduced TNF-α, nitric oxide, and lipid peroxidation, improved antioxidant enzyme activity, and attenuated injury to the liver, kidneys, and lungs (Edremitlioğlu et al., 2005; Lin et al., 2005; Oter et al., 2005; Chang et al., 2006; Yamanel et al., 2011; Yang et al., 2020; Lee et al., 2025). Pedoto et al. (2003) found that HBOT preconditioning suppressed inducible nitric oxide synthase and myeloperoxidase expression in lung tissue and attenuated LPS-induced hypotension and acidosis. In a zymosan-induced systemic inflammatory model, Luongo et al. (1998) reported that HBOT reduced TNF-α and nitrogen oxide levels and attenuated multiple-organ injury. These studies did not assess brain tissue, neurobehavioral outcomes, or cognitive function, but they indicate that HBOT can modulate inflammation, oxidative stress, and cellular injury under septic conditions. Direct animal studies of HBOT in SAE and representative supportive studies in systemic sepsis models are summarized in Table 1.

**Table 1:** Direct animal studies of HBOT in SAE and representative supportive studies in systemic sepsis models.

Evidence level	Study/model	HBOT protocol	Outcomes	Key findings	Relevance to SAE/limitations
Direct SAE evidence	Avtan et al. (2011); rat CLP model	100% O₂, 2.5 ATA; total session duration of 90 min, including 60 min of oxygen exposure at target pressure; administered at 1, 7, 13, and 19 h after CLP, for a total of four sessions	Cortical and hippocampal TNF-α, MDA, and GSH; Evans blue and HRP extravasation; occludin expression	Reduced cerebral MDA, restored GSH, and decreased Evans blue extravasation; no significant improvement in HRP extravasation or occludin expression; increased HRP extravasation in healthy rats	Directly assessed oxidative stress and BBB permeability in the septic brain, but different permeability measures produced inconsistent results, and neurobehavioral and cognitive outcomes were not evaluated
Direct SAE evidence	Wang Y. et al. (2024); male C57BL/6 J mouse CLP model	100% O₂, 1.9 ATA, 90 min per session; administered at 1 and 4 h after CLP, for a total of two sessions	HIF-1α and inflammatory mediators in blood and hippocampal tissue; microglial activation; Evans blue extravasation; Barnes maze and contextual fear conditioning	Reduced HIF-1α, TNF-α, IL-1β, and IL-6 levels; attenuated hippocampal microglial activation and BBB hyperpermeability; improved learning and memory; HIF-1α overexpression weakened these effects	Directly evaluated neuroinflammation, BBB permeability, and cognitive function in SAE, but HIF-1α was not manipulated in a specific cell type and the principal target cells remain unclear
Direct SAE evidence	Xie et al. (2026); male C57BL/6 J mouse CLP model	100% O₂, 0.20 MPa, 60 min per session; initiated on the day after CLP and administered once daily for 3, 7, or 14 days	Survival, murine sepsis score, SAE-associated neurobehavioral score, Morris water maze, hippocampal histopathology, and serum NSE and S100B	Improved neurobehavioral and learning and memory outcomes, attenuated hippocampal neuronal injury, and reduced NSE and S100B levels; some neurological and cognitive outcomes showed further improvement with longer treatment	Directly evaluated the effects of different HBOT treatment durations on neurobehavior, cognitive function, and brain injury in SAE, but the molecular mechanisms were not examined
Supportive systemic sepsis evidence	Buras et al. (2006); CLP model in C57BL/6 and IL-10-deficient mice	100% O₂; comparison of 1, 2.5, and 3 ATA and treatment every 12 or 24 h; the 2.5 ATA protocol administered every 12 h comprised eight sessions	Survival over 100 h, bacterial burden, IL-10 secretion by peritoneal macrophages, and treatment response in IL-10-deficient mice	Treatment at 2.5 ATA every 12 h improved survival, whereas 3 ATA reduced survival; HBOT increased IL-10 secretion by peritoneal macrophages, and its protective effect was lost in IL-10-deficient mice	Supports the involvement of IL-10 in the systemic protective effects of HBOT and indicates a pressure- and dose-dependent therapeutic window; brain and cognitive outcomes were not evaluated
Supportive systemic sepsis evidence	Bærnthsen et al. (2017); male Sprague–Dawley rat CLP model	100% O₂, 2.5 bar absolute, 90 min per session; administered at 24, 32, 48, 56, and 72 h after CLP, for a total of five sessions	Survival at 72 h and plasma IL-6, TNF-α, and IL-10 levels	Increased IL-10 at 48 h, with no significant changes in IL-6, TNF-α, or IL-10 at other time points; no significant improvement in 72-h survival	Provides a negative result for delayed HBOT and suggests that treatment timing may influence its effects; brain tissue, neurobehavioral outcomes, and cognitive function were not evaluated
Supportive systemic sepsis evidence	Halbach et al. (2019); male CD-1 mouse CLP model	98% O₂, 2.4 ATA, 60 min per session; single treatment at 1, 6, or 21 h after CLP, with additional repeated-treatment protocols at 1 + 6 h and 1 + 6 + 21 h	Survival at 72 h, bacterial burden, hepatic inflammatory mediators, and mitochondrial respiratory function	A single session at 1 h after CLP improved survival, whereas treatment at 6 or 21 h alone had no significant effect; repeated protocols that included the 1-h session did not further improve survival; bacterial burden and hepatic mitochondrial respiration were not significantly altered	Suggests an early therapeutic window for HBOT, but the study mainly assessed systemic survival and hepatic responses and included no SAE-specific outcomes
Supportive systemic sepsis evidence	Lin et al. (2005); Wistar–Kyoto rat model of LPS-induced endotoxemia	2 ATA, 60 min per session; administered at 1, 4, 9, and 24 h after LPS, for a total of four sessions	TNF-α, nitric oxide, superoxide, vascular reactivity, and mortality	Reduced TNF-α, nitric oxide, and superoxide levels, improved vascular reactivity, and reduced mortality	Supports the effects of HBOT on systemic inflammation, oxidative stress, and vascular function, but the study used an LPS-induced endotoxemia model and did not assess brain injury or cognitive function
Supportive systemic sepsis evidence	Yang et al. (2020); rat cecal slurry peritonitis model	100% O₂, 2.6 ATA, 90 min per session; three sessions within 24 h in the liver injury experiment and six sessions within 48 h in the survival experiment	Fourteen-day survival, inflammatory mediators, ROS, Akt/GSK-3β signaling, caspase-3, and hepatic injury	Improved 14-day survival, reduced inflammatory mediator and ROS levels, activated Akt/GSK-3β signaling, and decreased caspase-3 activation and hepatocyte apoptosis	Supports the regulation of sepsis-associated oxidative stress and cellular injury by HBOT, but the study mainly assessed hepatic outcomes

### 3.3 Indirect evidence from other neurological disorders

Because studies directly examining HBOT in SAE are limited, findings from traumatic brain injury, stroke, and Alzheimer’s disease provide additional evidence regarding its potential neuroprotective effects. These disorders differ from SAE in their underlying pathology but share features such as altered cerebral oxygen metabolism, neuroinflammation, oxidative stress, mitochondrial dysfunction, and cognitive impairment.

HBOT has been studied more extensively in traumatic brain injury and stroke. A systematic review and meta-analysis suggested that HBOT may improve selected neurocognitive outcomes in patients with traumatic brain injury, although the limited number and heterogeneity of the included studies prevent firm conclusions (Shahid et al., 2025). Studies involving patients with persistent post-concussion syndrome or severe traumatic brain injury reported improvements in cognitive and neurological function after HBOT, accompanied by changes in cerebral perfusion, angiogenesis, or biomarkers of neural injury (Tal et al., 2015; Li D. et al., 2025). In patients with chronic stroke, improvements in memory, attention, executive function, and activities of daily living have also been observed, with increased cerebral perfusion reported in some patients (Efrati et al., 2013; Hadanny et al., 2020). Animal studies further showed that HBOT suppressed p38-MAPK/CCL2- and TLR4/NF-κB-associated inflammatory responses, reduced neuronal apoptosis, and improved neurobehavioral and cognitive outcomes after brain injury (Hu et al., 2008; Liu et al., 2013; Yang Y. et al., 2014; Huang et al., 2016; Meng et al., 2016; Jiang B. et al., 2021). These findings suggest that HBOT may act by improving cerebral oxygenation, attenuating neuroinflammation, and promoting functional recovery after injury.

Studies of Alzheimer’s disease have mainly examined the effects of HBOT on chronic cognitive impairment, neurovascular function, and mitochondrial homeostasis. Some clinical studies reported improvements in cognitive scores and activities of daily living in patients with Alzheimer’s disease or amnestic mild cognitive impairment after HBOT, although the available studies have limitations in sample size and control-group design (Chen et al., 2020b; Lin et al., 2024). In APP/PS1 and 5 × FAD mice, HBOT improved learning and memory and attenuated associated neuropathological changes, accompanied by alterations in autophagy, mitochondrial quality control, neuroinflammation, and neurovascular function (Yang C. et al., 2024; Li B. et al., 2025; Yao et al., 2026). These processes partly overlap with mitochondrial injury, oxidative stress, and persistent neuroinflammation in SAE and therefore provide a mechanistic reference for the potential effects of HBOT on cognitive dysfunction. Overall, research in other neurological disorders supports the potential of HBOT to regulate cerebral oxygenation, inflammatory responses, mitochondrial homeostasis, and neurological recovery, although its effects in SAE require further investigation.

### 3.4 Overall assessment of the available evidence

The available evidence can be classified into three levels: direct animal studies of SAE, systemic sepsis models, and studies of other neurological disorders. Direct studies provide preliminary evidence that HBOT may attenuate oxidative brain injury, increased BBB permeability, and neuroinflammation and improve neurobehavioral and cognitive outcomes in animals with SAE (Avtan et al., 2011; Wang Y. et al., 2024; Xie et al., 2026). Studies of systemic sepsis and other neurological disorders further contribute to understanding the effects of HBOT on inflammatory regulation, redox homeostasis, mitochondrial function, and neural plasticity. Together, these findings constitute the current preclinical rationale for evaluating HBOT in SAE.

However, the available studies differ considerably in animal models, HBOT protocols, and outcome measures. The principal target cell types and signaling pathways affected by HBOT in SAE remain unclear. No study included in this review directly evaluated HBOT in patients with SAE. Current evidence therefore supports the potential of HBOT to modulate SAE-associated pathological processes but does not establish its precise mechanisms, optimal treatment protocol, or clinical efficacy. The levels of evidence and major limitations of the representative studies are summarized in Table 1. The available direct evidence and the proposed ferroptosis- and pyroptosis-related pathways through which HBOT may influence SAE are summarized in Figure 2.

**Figure 2:**
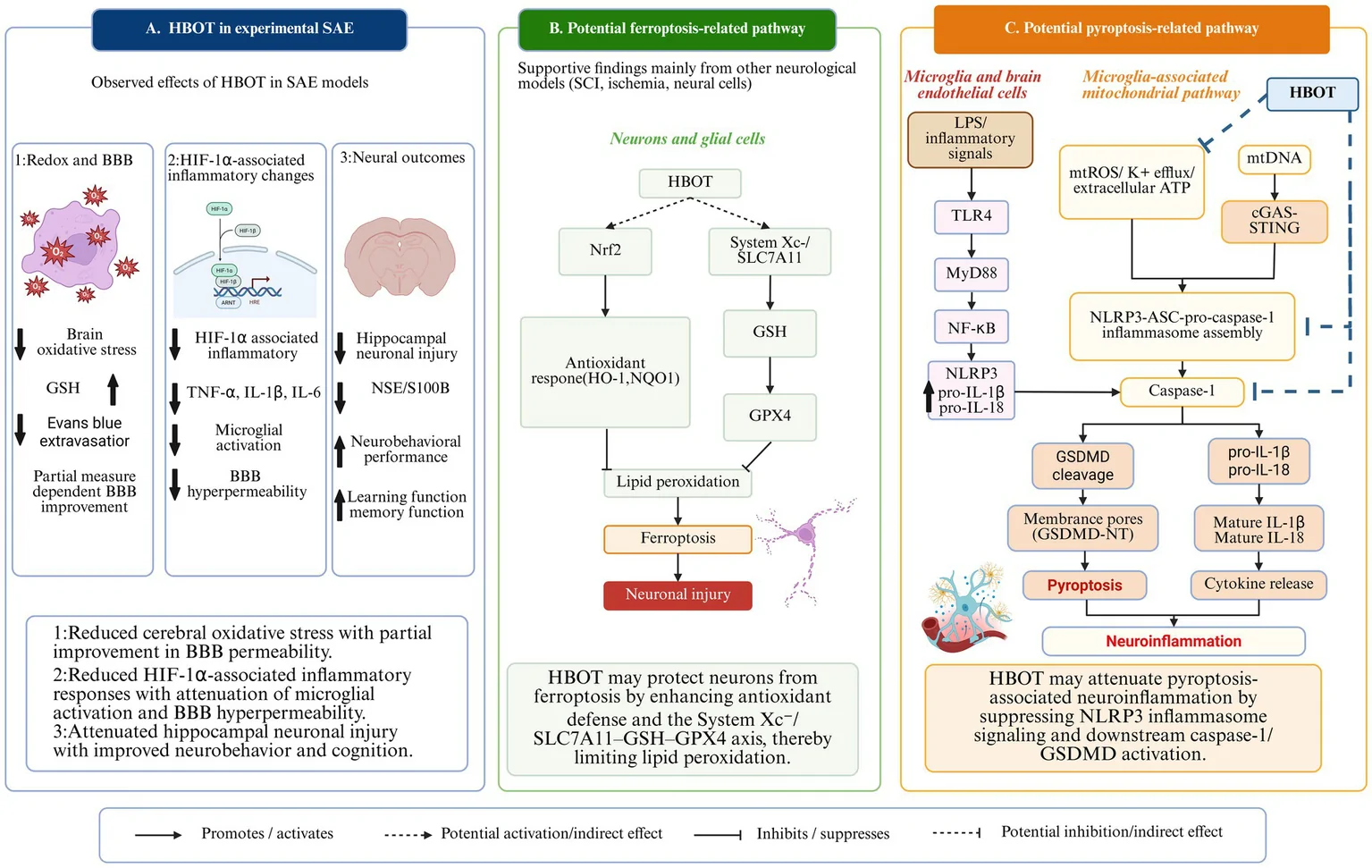
Direct evidence and potential molecular pathways through which hyperbaric oxygen therapy may modulate pathological processes in sepsis-associated encephalopathy. **(A)** Summarizes the observed effects of HBOT in experimental SAE models, including attenuation of oxidative stress, HIF-1α-associated inflammatory responses, microglial activation, BBB hyperpermeability, and neurobehavioral impairment. **(B)** Illustrates a potential ferroptosis-related pathway, mainly supported by studies in mechanistically relevant neurological injury models, in which HBOT may enhance antioxidant responses and the System Xc−/SLC7A11–glutathione (GSH)–glutathione peroxidase 4 (GPX4) axis, thereby limiting lipid peroxidation. **(C)** Illustrates a potential pyroptosis-related pathway in microglia and brain endothelial cells, in which HBOT may modulate mitochondrial reactive oxygen species (mtROS), NLRP3 inflammasome assembly, and downstream caspase-1/gasdermin D (GSDMD) activation. Solid arrows indicate promoting or activating effects; T-bars indicate inhibition or suppression; dashed arrows indicate potential or indirect effects. ATP, adenosine triphosphate; BBB, blood–brain barrier; cGAS, cyclic GMP–AMP synthase; GSH, glutathione; GSDMD, gasdermin D; HBOT, hyperbaric oxygen therapy; HIF-1α, hypoxia-inducible factor-1α; HO-1, heme oxygenase-1; IL, interleukin; LPS, lipopolysaccharide; mtDNA, mitochondrial DNA; mtROS, mitochondrial reactive oxygen species; NLRP3, NOD-like receptor family pyrin domain-containing 3; NQO1, NAD(P)H quinone dehydrogenase 1; Nrf2, nuclear factor erythroid 2-related factor 2; SAE, sepsis-associated encephalopathy; SLC7A11, solute carrier family 7 member 11; STING, stimulator of interferon genes; TNF-α, tumor necrosis factor-α. Created in BioRender. Tu, F. (2026) https://BioRender.com/q4x1qxt

## 4 Conclusions and perspectives

SAE is a serious neurological complication of sepsis. Its complex pathogenesis involves interactions among BBB disruption, neuroinflammation, microglial activation, oxidative stress, ferroptosis, and pyroptosis, ultimately leading to neuronal injury and cognitive dysfunction. Within this multifactorial and multilevel pathological process, oxidative stress and ROS accumulation represent important links among these mechanisms. Excessive ROS not only disrupt cellular redox homeostasis but also promote ferroptosis through membrane lipid peroxidation and contribute to pyroptosis through mitochondrial injury and NLRP3 inflammasome activation, thereby further aggravating neuroinflammation. ROS and inflammatory mediators can also damage the BBB and facilitate the entry of peripheral inflammatory mediators and other harmful substances into the brain, resulting in further neuronal injury and cognitive impairment. Excessive ROS production therefore links BBB disruption, neuroinflammation, ferroptosis, and pyroptosis and may represent a potential therapeutic target.

HBOT increases tissue oxygen tension and may improve microcirculation and mitochondrial function, with several animal studies reporting neuroprotective effects. Available evidence suggests that HBOT may attenuate neuroinflammation and cognitive impairment by improving BBB homeostasis and regulating redox balance and microglial inflammatory responses. Findings from other neurological injury models further indicate that ferroptosis- and pyroptosis-related pathways may contribute to its neuroprotective effects. Animal studies of SAE have also reported improvements in neurobehavioral performance, learning and memory, and selected markers of neural injury after HBOT. These findings suggest that HBOT may attenuate SAE-associated brain injury by acting on several interconnected pathological processes. However, the current evidence is derived mainly from animal experiments, and HBOT protocols and outcome measures differ considerably among studies. No study included in this review directly evaluated HBOT in patients with SAE.

Future studies should further investigate the mechanisms through which HBOT affects SAE. Mechanistic studies should systematically evaluate its regulation of BBB injury, oxidative stress, and regulated cell death and determine how these effects contribute to neuronal protection and cognitive improvement. Randomized controlled trials are also needed to evaluate the effects of HBOT on cognitive function, biomarkers of neurological injury, survival, and other clinical outcomes in patients with SAE. Integration of animal and clinical studies may provide more robust evidence regarding the safety and efficacy of HBOT in SAE and support its clinical translation.

Overall, HBOT represents a non-pharmacological intervention worthy of further evaluation in SAE, although its precise mechanisms, appropriate treatment protocols, and clinical value remain to be established.
